# Cost-utility analysis of community-based interventions for hypertension control in Vietnam

**DOI:** 10.21203/rs.3.rs-4328156/v1

**Published:** 2024-05-07

**Authors:** Viet Nguyen, Duc Anh Ha, Oanh Mai Tran, Hoa L. Nguyen, Robert J Goldberg, Jeroan J. Allison, Neil S. Fleming, Phuong Khanh Nguyen

**Affiliations:** Ministry of Health; Ministry of Health; Ministry of Health; University of Massachusetts Chan Medical School; University of Massachusetts Chan Medical School; University of Massachusetts Chan Medical School; Baylor University; Ministry of Health

**Keywords:** community-based intervention, hypertension, cost-utility, cost-effective, storytelling, Vietnam

## Abstract

Between 2010 and 2011, stakeholders implemented a multi-faceted community-based intervention in response to the escalating issue of uncontrolled hypertension in Hung Yen province, Vietnam. This initiative integrated expanded community health worker services, home blood pressure self-monitoring, and a unique “storytelling intervention” into routine clinical care. From the limited societal perspective, our study evaluates the cost-effectiveness of this intervention using a Markov model with a one-year cycle over a lifetime horizon. The analysis, based on a cohort of 671 patients, reveals a lifetime incremental cost of approximately VND 90.37 million (USD 3,930) per quality-adjusted life year (QALY) gained. With a willingness to pay at three times GDP (VND 259.2 million per QALY), the intervention proves cost-effective 80% of the time. This research underscores the potential of the community-based approach to effectively control hypertension, offering valuable insights into its broader implications for public health.

## Introduction

Cardiovascular disease (CVD) is the leading cause of death in Vietnam, accounting for 33.18% of all deaths in 2017 [1]. Major risk factors for CVD, including hypertension, diabetes, unhealthy dietary practices, and overweight/obesity are either on the rise or at alarming levels in Vietnam [2–4]. National data show a high prevalence of hypertension (HTN) in communities throughout Vietnam and the general population consumes high levels of sodium in their diet [4].

With the desire to control hypertension in Vietnam, the program “Conquering Hypertension in Vietnam: Solutions from Grassroots Health” was developed [5]. Between 2020 and 2021, a cluster randomized controlled trial (RCT) of a community-based intervention included three carefully selected enhancements integrated into routine clinical care: (1) expanded community health worker services, (2) home blood pressure self-monitoring, and (3) a “storytelling intervention” was conducted in Hung Yen province, Vietnam [5]. In sixteen communities, 671 adult men and women with uncontrolled hypertension were randomly allocated to either an intervention or a comparison group to evaluate the effectiveness of the multi-faceted intervention on the control of elevated blood pressure.

In parallel with the evaluation of the effectiveness of the hypertension control model in the community, the cost-effectiveness of the model was assessed for purposes of providing information to policymakers for the possible nationwide dissemination of the multi-component intervention program. The objectives of the present study were to carry out a cost-utility analysis of this community-based intervention with quality adjusted life years being the principal study outcome.

## Methods

**Figure 1:**
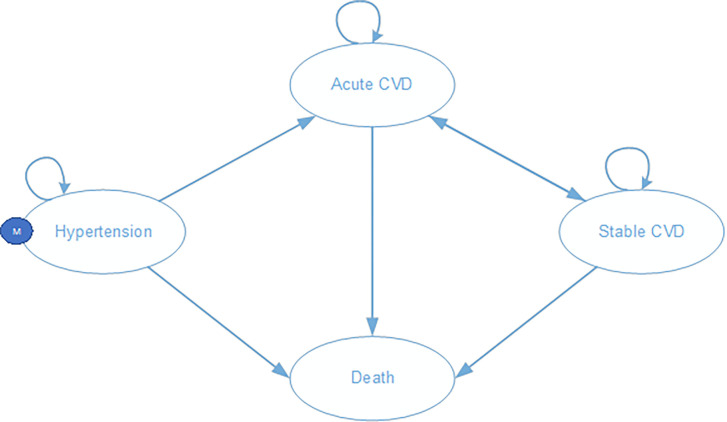
Different health states in the Markov model Notes: CVD: cardiovascular disease. Patients start in the initial hypertension state. Patients can remain in this state or move to either acute CVD or death. From the acute CVD state, patients can move to stable CVD or death state, or may experience recurrent CVD events. From the stable CVD state, patients may stay in the same health state, or they may have a recurrence of CVD or can move to death.

A Markov model with a 1-year cycle length throughout a lifetime horizon was developed to measure the incremental cost-utility of the community-based intervention [6]. In the Markov model, patients start at age of 50 in their initial hypertension state. Patients can remain in this state or move to either the development of an acute cardiovascular event or death. The cardiovascular (CVD) events included the development of either a myocardial infarction (ICD-10 code I21) or a cerebrovascular disease (ICD-10 codes I60 to I66). At the end of each cycle in the acute CVD state, patients can move to either stable CVD or death, or may experience recurrent CVD events and then stay in the same state. In the stable CVD state, patients can experience death or stay in the same health state, or they may have a recurrence of CVD and move to acute CVD. Figure 1 illustrates the complete model.

### Transition probabilities

In the community-based randomized controlled trial, 671 patients with uncontrolled hypertension were randomly allocated to either intervention or comparison group status [5]. Changes in blood pressure (BP) and data on age, sex, and smoking status were collected in both groups 12 months after trial enrollment. A cardiovascular risk prediction tool designed for the Asian population was employed to predict the annual acute CVD risk for this patient population [7].

The likelihood of recurrent CVD events in patients at the conclusion of their acute or stable state of CVD was anticipated to be higher than in individuals without a history of CVD. However, due to the absence of an appropriate equation or data to estimate this probability for these individuals, it was assumed that the annual transition probability for patients with a history of CVD was the same as for those without CVD, since they were of the same age and sex.

Data from the Vietnam Life Table 2016 were utilized to quantify the transition probability from any health state to the risk of dying from any cause, assumed to be the same as the transition probability of hypertensive patients to death [8]. The mortality rate escalation in patients in varying states of CVD was gathered from a prior study conducted in Vietnam [9].

### Costs

Costs were evaluated from the limited societal perspective [10]. Parameters related to intervention costs were collected from the RCT [5]. The intervention costs encompassed design, implementation, and monitoring and evaluation expenses. The overall cost of intervention per patient is Vietnam Dong (VND) 4 million annually, corresponding to 174 U.S. dollars. If the multi-component intervention was to be implemented on a national scale, the design, monitoring, and evaluation costs can be eliminated and only the cost of implementing the intervention, about VND 430 thousand/person/year (19 U.S. dollars), should be considered.

Data on treatment costs for patients with hypertension, acute CVD, and stable CVD were collected from a database of six Vietnamese hospitals, including one central hospital in Hanoi, and one provincial hospital, four district hospitals in Hung Yen province for patients with the relevant ICD codes. The dataset comprised a total of 15,533 outpatient records and 2,553 inpatient records. In addition to the medical costs, non-medical costs and frequency of treatment were also collected from interviews of 178 patients who were receiving treatment at those hospitals. All costs were standardized to the value for the year 2021. The discount rate for costs was 3% in the base case. Details are provided in table 1.

### Health utilities

Quality of life (utilities) for hypertensive, acute CVD, and stable CVD cases were applied in the model. These data were also collected from the patient interviews. Health utility of states was measured by EQ-5D-5L in Vietnam [11]. The discount rate for effects was also 3% in the base case according to published guidelines [12, 13].

### Analysis

Cost-effectiveness was assessed through deterministic and sensitivity analyses. One-way sensitivity and probabilistic sensitivity analyses were carried out to identify the key parameters influencing cost-effectiveness outcomes and to quantify the overall uncertainty associated with the results across all input parameters [14]. For the one-way sensitivity analysis, a single parameter was varied between a low to high range based on the 95% confidence interval of the parameter estimate where available.

With regards to the probabilistic sensitivity analysis, the incremental cost-effectiveness ratio (ICER) was repeatedly calculated 10,000 times by simultaneously varying the values of all input parameters based on probability distributions [15]. Results are presented on a cost-effectiveness plane and an acceptability curve. The cost-effectiveness threshold of 3 GDP per QALY gained in 2021 (VND 259.2 million ~ USD 11,269) [16] will be used as recommended by the World Health Organization [17].

### Ethical statement

This randomized trial was approved by the Institutional Review Board at the Health Strategy and Policy Institute (HSPI) in Hanoi, Vietnam (Decision 171/QD-CLCSYT on September 10, 2019). Written informed consent was obtained from all patients. This trial was registered at ClinicalTrials.gov (Registration number: NCT03590691, registration date May 31, 2018).

## Results

The results pertaining to the number of QALYs gained, incremental costs, and incremental cost-effectiveness ratios for the full-time horizon model are presented in Table 2. Applying a discount rate of 3% for both cost and effectiveness, the total cost was VND 260.38 million for the intervention group and VND 258.48 million for the control group. The intervention group exhibited higher effectiveness compared to the control group, showing an increased life year of 0.02 and an increased QALY of 0.05. Consequently, the ICER was calculated as 90.37 million VND per Life Year (LY) and 35.14 million VND per QALY. Importantly, this incremental cost per QALY gained remains well below the threshold of 1 GDP per capita of Vietnam in 2021, which is USD 3,756, equivalent to nearly VND 86.4 million; representing a cost-effective intervention.

### Sensitivity analysis

**Figure 2:**
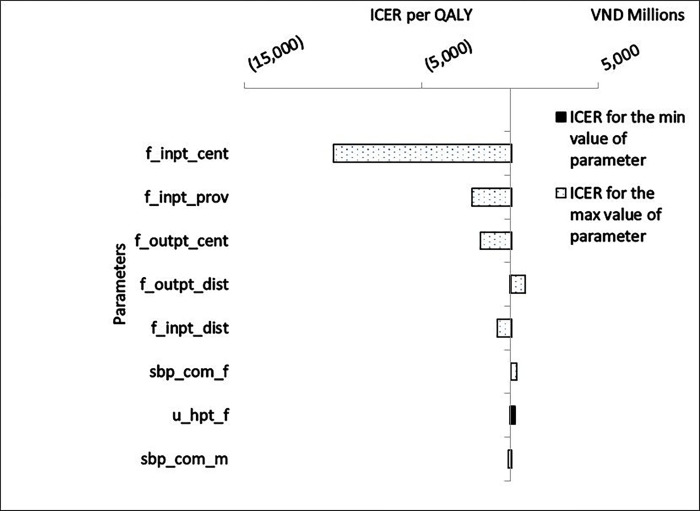
Tornado diagram for univariate sensitivity analysis Note: f_inpt_cent: Frequency of central inpatient treatment 1 year; f_inpt_prov: Frequency of inpatient treatment at provincial level 1 year; f_outpt_cent: Frequency of central outpatient treatment 1 year; f_outpt_dist: Frequency of outpatient treatment at district level 1 year; f_inpt_dist: Frequency of inpatient treatment at district level 1 year; sbp_com_f: systolic blood pressure (SBP) of women in the control group; u_hpt_f: Utility in female hypertensive patients; sbp_com_m: SBP of men in the control group

The univariate sensitivity analysis demonstrated that the parameter group of treatment frequency exerted the most significant influence on the present results. In particular, the frequency of inpatient treatment at the central hospital level emerged as the most impactful parameter; as this frequency increased, there was a potential for cost savings of VND 10 billion per QALY in the intervention. The frequencies of inpatient treatment at the provincial level and outpatient treatment at the central level demonstrated a similar impact but of a lower magnitude. On the other hand, an increase in the frequency of outpatient treatment at the district level resulted in a slight increase in the ICER per QALY. Following the beneficial impact of the parameter of treatment frequency, was the parameter group on the effectiveness of the intervention such as the SBP index and the utility in female hypertensive patients, but their magnitudes were not significant on the principal study outcome (Figure 2).

**Figure 3:**
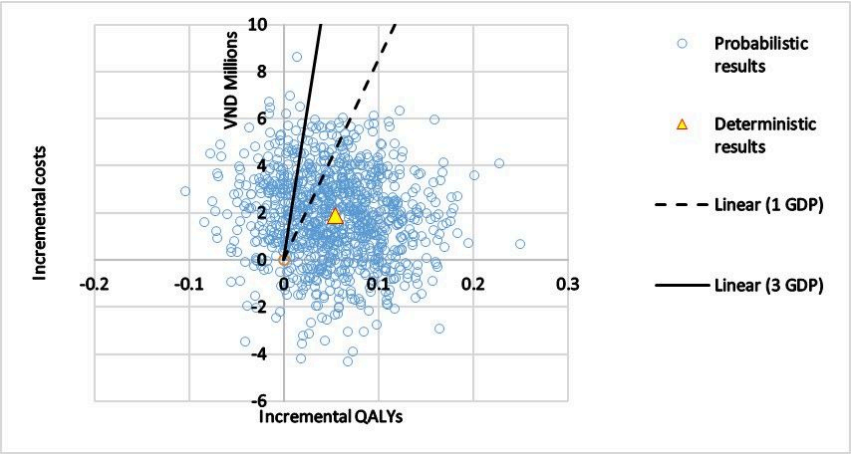
Probabilistic analysis: Incremental Costs vs. Incremental QALYs Notes: QALYs: quality-adjusted life years; 1 Gross Domestic Product (GDP) per capita in 2021: VND 86.4 million (USD 3,756); 3 GDP: VND 259.2 million

The results of the probability sensitivity analysis of 10,000 simulations are presented in figure 3, with the blue circles representing the resulting ICERs. The green line represents the willingness to pay 1 GDP while the orange line represents the willingness to pay 3 GDP. The main part of the “cloud” remains to the right of the 1–3 GDP threshold and suggests that the intervention has a high probability to be cost-effective.

**Figure 4:**
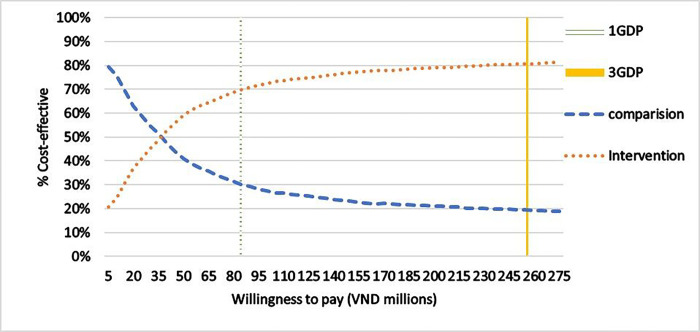
Acceptability curve Notes: 1 Gross Domestic Product (GDP) per capita in 2021: VND 86.4 million (USD 3,756); 3 GDP: VND 259.2 million

Analysis of the willingness to pay threshold shows that if the threshold of willingness to pay would be below VND 35 million, the intervention had a lower probability to be cost-effective, below 50%, compared to the control group. However, at the willingness to pay of 1 GDP in 2021, USD 3,756 equivalent to about VND 86.4 million, the cost-effective rate increased to 70%. This rate would increase to more than 80% when the willingness to pay was at 3 GDP per capita, equivalent to approximately VND 259.2 million (Figure 4).

## Discussion

We performed a cost-effectiveness analysis of an intervention model to control elevated BP findings among adults with hypertension in the community, including training for village health workers to enhance the ability of patients to change their lifestyle habits and self-monitoring of their BP. The deterministic analysis showed that the intervention increased VND 90.37 million per LY gained and VND 35.14 million per QALY gained. This incremental cost per QALY is below the threshold of 1 GDP per capita and, after use of a probabilistic analysis, the intervention results in a cost-effectiveness ratio of more than 80% at the threshold of 3 GDP per capita.

Since the intervention model was designed according to the local context, it is difficult to compare the present results with other cost-effectiveness studies. However, a systematic review that was published in 2017 [18] of the economic evidence for hypertension interventions in the community concluded that most studies were cost-effective or cost-saving. This review included 34 published articles (16 from the US and 18 from other countries) that examined the impact of 25 educational interventions, 3 BP self-monitoring interventions, and 6 screening interventions. The incremental costs for a 1 mmHg decrease in systolic blood pressure averaged USD 62 (range 40–114) and USD 13,986 (range 6,683–58,610) for an additional year of life.

### Study strengths and limitations

We selected the simplest Markov model for grouping cardiovascular complications into acute CVD and stable CVD states for purposes of evaluating the effectiveness of interventions to control hypertension in the community; assessment of the effectiveness of treating the complications associated with CVD was not examined. However, this approach also reduced the complexity of the model when reducing the model’s calculation errors and reducing the burden of collecting transitional probability parameters, treatment related costs, and treatment effectiveness.

Moreover, in addressing concerns regarding the cost data gathered amidst the Covid-19 pandemic, sensitivity analysis was conducted. The findings indicate that the parameter of outpatient treatment frequency at the district level slightly influences the incremental cost-effectiveness ratio (ICER) per quality-adjusted life year (QALY). This observation would be attributed to the intervention’s ability to modify the disease state, decrease the probability of cardiovascular disease-related complications, and enhance the proportion of patients effectively managing hypertension through outpatient treatment at the district level.

The study used a cardiovascular risk prediction tool for Asian population [7] to extrapolate the effectiveness of the intervention to the reduced number of complications and the final outputs were the number of life years and QALY gained. The risk prediction tool was considered the most suitable for use with relatively simple input variable requirements including systolic blood pressure index, age, sex, total cholesterol data and smoking status. However, total cholesterol data were not available in our study for factoring into the calculation. We expected that the total cholesterol index would exhibit positive changes in the intervention group and this limitation would not affect the conclusions of this study. In the absence of the cholesterol index, estimate of the intervention’s cost-effectiveness can be considered as conservative.

## Conclusion

The multi-faceted community-based intervention would have a high probability to be cost-effective for hypertensive patients over a longer time duration and to be disseminated on a national basis. As is often the case in public health studies, preventive interventions need several years for both the actual intervention and its effect on long-term outcomes to be examined. Ability to follow the intervention over many years and patient follow-up also allow the interventions to be tailored with greater probability of effectiveness as well as compliance.

## Figures and Tables

**Table 1.** Input parameters

Variables	Distribution	Mean	Unit	Standard error	Source
SBP of men in the intervention group	Normal	147	mmHg	1.102	RCT [5]
SBP of women in the intervention group	Normal	141	mmHg	1.042	RCT [5]
Smoking rate among men in the intervention group	Beta	0.228		0.023	RCT [5]
Smoking rate among women in the intervention group	Beta	0.006		0.001	RCT [5]
SBP of men in the control group	Normal	154	mmHg	1.673	RCT [5]
SBP of women in the control group	Normal	159	mmHg	1.558	RCT [5]
Smoking rate among men in the control group	Beta	0.145		0.015	RCT [5]
Smoking rate among women in the control group	Fixed	0		0.000	RCT [5]
Mortality rate from CVD among men	Beta	0.003		0.001	Minh et al. 2006 [9]
Mortality rate from CVD among women	Beta	0.002		0.000	Minh et al. 2006 [9]
Intervention costs - Design - 1 year	Gamma	335,294	VND	67,059	RCT [5]
Intervention costs - Implementation - 1 year	Gamma	426,471	VND	85,294	RCT [5]
Intervention costs - monitoring & evaluation- 1 year	Gamma	3,238,235	VND	647,647	RCT [5]
Cost of acute CVD treatment at central level per admission	Gamma	51,652,576	VND	9,879,613	Hospital database
Cost of acute CVD treatment at provincial level per admission	Gamma	9,623,739	VND	448,833	Hospital database
Cost of acute CVD treatment at district level per admission	Gamma	1,725,492	VND	113,419	Hospital database
Cost of stable CVD treatment at central level per visit	Gamma	785,000	VND	343,566	Hospital database
Cost of stable CVD treatment at provincial level per visit	Gamma	562,580	VND	2,679	Hospital database
Cost of hypertension treatment at provincial level per visit	Gamma	475,225	VND	4,601	Hospital database
Cost of hypertension treatment at district level per visit	Gamma	316,417	VND	1,906	Hospital database
Travel expenses per inpatient admission	Gamma	1,047,979	VND	225,224	Patient interview
Travel expenses per outpatient visit	Gamma	46,477	VND	11,653	Patient interview
Cost of meals per inpatient admission	Gamma	635,947	VND	44,933	Patient interview
Cost of meals per outpatient visit	Gamma	6,898	VND	1,220	Patient interview
Other costs per inpatient admission	Gamma	454,905	VND	108,018	Patient interview
Other costs per outpatient visit	Gamma	75,880	VND	15,638	Patient interview
Frequency of central inpatient treatment 1 year	Normal	1.39		0.05	Patient interview
Frequency of inpatient treatment at provincial level 1 year	Normal	1.36		0.19	Patient interview
Frequency of inpatient treatment at district level 1 year	Normal	0.39		0.05	Patient interview
Frequency of central outpatient treatment 1 year	Normal	1.70		0.29	Patient interview
Frequency of outpatient treatment at provincial level 1 year	Normal	5.86		0.39	Patient interview
Frequency of outpatient treatment at district level 1 year	Normal	7.50		0.36	Patient interview
Utility in male hypertensive patients	Beta	0.84		0.05	Patient interview
Utility in female hypertensive patients	Beta	0.64		0.04	Patient interview
Utility in male stable CVD patients	Beta	0.73		0.06	Patient interview
Utility in female stable CVD patients	Beta	0.64		0.06	Patient interview
Utility in male acute CVD patients	Beta	0.43		0.08	Patient interview
Utility in female acute CVD patients	Beta	0.47		0.07	Patient interview
Effect discount rate	Fixed	3%			Guidelines [12, 13]
Cost discount rate	Fixed	3%			Guidelines [12, 13]

Notes: SBP: systolic blood pressure; CVD: cardiovascular disease. The data were stratified by sex to align with the criteria of the risk prediction tool

**Table 2.** Deterministic analysis

	Comparison	Intervention
Cost (VND)	258,480,291	260,382,154
LYs	33.23	33.25
QALYs	24.16	24.21
Incremental cost		1,901,863
Incremental LY		0.02
Incremental QALY		0.05
ICER/LY (VND/LY)		90,367,969
ICER/QALY (VND/QALY)		35,134,939

Notes: LYs: life years; QALYs: quality-adjusted life years; ICER: incremental cost-effectiveness ratio

## Key Message

Our study demonstrates that the multi-faceted community-based intervention for hypertensive patients has a high likelihood of being cost-effective over a longer duration. The findings suggest that sustained implementation and follow-up over several years can enhance the effectiveness and compliance of interventions. This supports the recommendation for the nationwide dissemination of the intervention, emphasizing the importance of long-term commitment and investment in public health strategies.

## Abbreviations

Markov Model: A mathematical model used to analyze the transition of individuals between different health states over time.
ICD-10: International Statistical Classification of Diseases and Related Health Problems, 10th Revision; a medical classification list by the World Health Organization (WHO).
CVD: Cardiovascular Disease; a class of diseases that involve the heart or blood vessels, including myocardial infarction (heart attack) and cerebrovascular disease (stroke).
BP: Blood Pressure; the pressure of circulating blood against the walls of blood vessels.
VND: Vietnamese Dong; the currency of Vietnam.
USD: United States Dollar; the currency of the United States.
EQ-5D-5L: EuroQol Five-Dimension Five-Level; a standardized instrument for measuring health-related quality of life.
GDP: Gross Domestic Product; a monetary measure of the market value of all final goods and services produced in a period by a country.
QALY: Quality-Adjusted Life Year; a measure of disease burden, including both the quality and the quantity of life lived.

## References

[1] Statista Research Department. Main fatal non-communicable diseases based on percentage of total deaths in Vietnam in 2017, by type 2023 [https://www.statista.com/statistics/1107626/vietnam-main-fatal-non-communicable-diseases/.

[2] Nguyen TT, Hoang MV. Non-communicable diseases, food and nutrition in Vietnam from 1975 to 2015: the burden and national response. Asia Pac J Clin Nutr. 2018;27(1):19–28. 29222878 10.6133/apjcn.032017.13

[3] Son PT, Quang NN, Viet NL, Khai PG, Wall S, Weinehall L, Prevalence, awareness, treatment and control of hypertension in Vietnam-results from a national survey. J Hum Hypertens. 2012;26(4):268–80. 21368775 10.1038/jhh.2011.18

[4] Bao TQ, Hoang VM, Lan VH, Linh BP, Giang KB, Nga PQ Risk factors for Non-Communicable Diseases among adults in Vietnam: Findings from the Vietnam STEPS Survey 2015. J Global Health Sci. 2020;2(1).

[5] Ha DA, Tran OT, Nguyen HL, Chiriboga G, Goldberg RJ, Phan VH, Conquering hypertension in Vietnam-solutions at grassroots level: study protocol of a cluster randomized controlled trial. Trials. 2020;21(1):985. 33246495 10.1186/s13063-020-04917-8 PMC7694904

[6] Nguyen TP, Wright EP, Nguyen TT, Schuiling-Veninga CC, Bijlsma MJ, Nguyen TB, Cost-Effectiveness Analysis of Screening for and Managing Identified Hypertension for Cardiovascular Disease Prevention in Vietnam. PLoS ONE. 2016;11(5):e0155699. 27192051 10.1371/journal.pone.0155699 PMC4871542

[7] Asia Pacific Cohort, Studies C, Barzi F, Patel A, Gu D, Sritara P, Lam TH, Cardiovascular risk prediction tools for populations in Asia. J Epidemiol Community Health. 2007;61(2):115–21. 17234869 10.1136/jech.2005.044842 PMC2465638

[8] General statistics office. Major findings: the 1/4/2015 time-point population change and family planning survey. Statistical publishing house 2016.

[9] Minh HV, Byass P, Wall S. Mortality from cardiovascular diseases in Bavi District, Vietnam. Scand J Public Health Suppl. 2003;62:26–31. 14649634 10.1080/14034950310015077

[10] Kim DD, Silver MC, Kunst N, Cohen JT, Ollendorf DA, Neumann PJ. Perspective and Costing in Cost-Effectiveness Analysis, 1974–2018. PharmacoEconomics. 2020;38(10):1135–45. 32696192 10.1007/s40273-020-00942-2 PMC7373843

[11] Mai VQ, Sun S, Minh HV, Luo N, Giang KB, Lindholm L, An EQ-5D-5L Value Set for Vietnam. Qual Life Res. 2020;29(7):1923–33. 32221805 10.1007/s11136-020-02469-7 PMC7295839

[12] Khorasani E, Davari M, Kebriaeezadeh A, Fatemi F, Akbari Sari A, Varahrami V. A comprehensive review of official discount rates in guidelines of health economic evaluations over time: the trends and roots. (1618–7601 (Electronic)). 10.1007/s10198-022-01445-x 35235078

[13] Vietnam Ministry of Health. Guidelines for health technology assessment reports (draft version in 7.2023). In: Department of Health Insurance MoH, editor.; 2023.

[14] York. Sensitivity Analysis [online] York Health Economics Consortium 2016 [https://yhec.co.uk/glossary/sensitivity-analysis/.

[15] York P. /Stochastic Sensitivity Analysis [online]: York Health Economics Consortium; 2016 [https://yhec.co.uk/glossary/probabilistic-stochastic-sensitivity-analysis/.

[16] World Bank. Vietnam [Data Commons - Place Explorer] 2022 [https://datacommons.org/place/country/VNM.

[17] Marseille E, Larson B, Kazi DS, Kahn JG, Rosen S. Thresholds for the cost-effectiveness of interventions: alternative approaches. Bull World Health Organ. 2015;93(2):118–24. 25883405 10.2471/BLT.14.138206 PMC4339959

[18] Zhang D, Wang G, Joo H. A Systematic Review of Economic Evidence on Community Hypertension Interventions. Am J Prev Med. 2017;53(6S2):S121–30. 29153113 10.1016/j.amepre.2017.05.008 PMC5819001

